# Computational determination of toxicity risks associated with a selection of approved drugs having demonstrated activity against COVID-19

**DOI:** 10.1186/s40360-021-00519-5

**Published:** 2021-10-21

**Authors:** Maral Aminpour, Williams Ernesto Miranda Delgado, Soren Wacker, Sergey Noskov, Michael Houghton, D. Lorne J. Tyrrell, Jack A. Tuszynski

**Affiliations:** 1 Department of Biomedical Engineering, University of Alberta, Edmonton, AB T6G 1Z2 Canada; 2 Centre for Molecular Simulation, Department of Biological Sciences, University of Calgary, 2500 University Drive, Calgary, AB T2N 1N4 Canada; 3 Department of Medical Microbiology & Immunology, 6-010 Katz Group-Rexall Centre for Health Research, Li Ka Shing Institute of Virology, University of Alberta, Edmonton, AB T6G 2E1 Canada

**Keywords:** COVID-19, Toxicity, Repurposing drugs, hERG)

## Abstract

**Background:**

The emergence and rapid spread of SARS-CoV-2 (severe acute respiratory syndrome coronavirus 2) in thelate 2019 has caused a devastating global pandemic of the severe pneumonia-like disease coronavirus disease 2019 (COVID-19). Although vaccines have been and are being developed, they are not accessible to everyone and not everyone can receive these vaccines. Also, it typically takes more than 10 years until a new therapeutic agent is approved for usage. Therefore, repurposing of known drugs can lend itself well as a key approach for significantly expediting the development of new therapies for COVID-19.

**Methods:**

We have incorporated machine learning-based computational tools and in silico models into the drug discovery process to predict Adsorption, Distribution, Metabolism, Excretion, and Toxicity (ADMET) profiles of 90 potential drugs for COVID-19 treatment identified from two independent studies mainly with the purpose of mitigating late-phase failures because of inferior pharmacokinetics and toxicity.

**Results:**

Here, we summarize the cardiotoxicity and general toxicity profiles of 90 potential drugs for COVID-19 treatment and outline the risks of repurposing and propose a stratification of patients accordingly. We shortlist a total of five compounds based on their non-toxic properties.

**Conclusion:**

In summary, this manuscript aims to provide a potentially useful source of essential knowledge on toxicity assessment of 90 compounds for healthcare practitioners and researchers to find off-label alternatives for the treatment for COVID-19. The majority of the molecules discussed in this manuscript have already moved into clinical trials and thus their known pharmacological and human safety profiles are expected to facilitate a fast track preclinical and clinical assessment for treating COVID-19.

**Supplementary Information:**

The online version contains supplementary material available at 10.1186/s40360-021-00519-5.

## Background

Since December 2019, much of the world has suffered from the outbreak of coronavirus disease 2019 (COVID-19), the disease caused by a novel human coronavirus, severe acute respiratory syndrome coronavirus 2 (SARS- CoV-2) [1]. The WHO (World Health Organization) proclaimed the outbreak of coronavirus disease-2019 (COVID-19) to be a Public Health Emergency of International Concern (PHEIC), which was the highest level of epidemic prevention in the world, suggesting its gravity [2]. As of now, nearly 189 million people worldwide have been infected with SARS-CoV2, with almost 4.1 million fatalities by July 14, 2021. SARS-CoV-2 genome is an enveloped, non-segmented positive-sense RNA β-coronavirus, associated with the viruses that originated the SARS and MERS (Middle East respiratory syndrome) outbreaks since early 2000s and 2012, respectively. One of the crucial threats of COVID-19 is the combination of respiratory failure and cardiovascular complications combined with widespread endothelial dysfunction and severe inflammation. It appears that an overproduction of pro-inflammatory cytokines (known as “cytokine storm”) that can be detected with interleukins and tumor necrosis biomarkers, is observed in the lungs of severely ill COVID-19 patients [3]. Coronaviruses have spherical or pleiomorphic shape with size approximately 80 to 160 nm in diameter. Coronaviruses free their nucleocapsid into the host cell by fusing their envelope with the host cell membrane. The spike glycoprotein (S) arbitrates the entrance of the virus and is the major factor of cell tropism and pathogenesis. SARS-CoV-2 spike proteins bind to ACE2 receptor proteins on the host cell surface, perceived as angiotensin converting enzyme 2 (ACE2).

The high morbidity and mortality rates were ascribed to the lack of effective drug treatment. COVID-19, for which SARS-CoV-2 is the etiological agent, poses a serious threat to human life during the continuation of the global outbreak. Although vaccines have been developed recently, they are not accessible to everyone and not everyone can receive these vaccines. Also, with new variants of COVID emerging, vaccines may not offer 100% protection. Emergence of new strains of SARS-COV-2 is another hurdle on the way of the vaccines and tailoring of available vaccines narrowly to the new mutants might be needed to adopt and make them effective. Therefore, the search for efficacious therapeutic agents to treat COVID-19 patients is vital and urgent. Novel approaches to drug design and discovery are being utilized to explore therapeutic drug candidates for COVID-19. Identification of effective therapeutics/pharmacological treatments with very little time available for the new drug discovery is a unique challenge for health professionals to tackle the unprecedent spread of SARS-CoV-2 disease. Currently, specific therapeutic agents or antiviral compounds are still being investigated for treatment against SARS-CoV-2.

Developing a new prescription medicine that warrants marketing approval may take more than 10 to 15 years and cost close to a billion dollars with a success rate of only 2%. It is, therefore, more time- and cost-effective to develop new therapies by at least initially probing the existing antiviral and other drug databases against SARS-CoV2 molecular targets [4, 5]. Hence, reassessing the efficacy and safety of licensed and experimental drugs has become the primary alternative recommended by the WHO and other health agencies to tackle emerging health crises. In the past, repurposing strategy has been beneficial for identifying potential therapeutic compounds against numerous viral diseases with high risk of death such as Zika, Ebola and hepatitis C viral infections to name a few [6–8].

A potential drug candidate should have unobjectionable pharmacokinetic and pharmacodynamic profiles, along with a high safety margin with low risks of toxicity and side effects. The purpose of this paper is to computationally assess the toxicity risks associated with a selection of approved drugs having demonstrated activity against the COVID-19 virus based on two previously published independent studies and our intention is to rank the risks of repurposing these drugs and to recommend a stratification of patients according to these risks [9, 10]. The first study includes 21 chemical substances (antibacterial, antimalarial and antiparasitic agents, and other drugs) that have been deemed applicable for experimental therapies, mainly for symptomatic treatment, even though they were originally selected to primarily target the underlying cause [9]. The second study involves 69 drugs (FDA-approved drugs, drugs in clinical trials and/or preclinical compounds) that have been identified by combining a systematic chemoinformatic drug search and a pathway-centric analysis that targets parts of the resulting network [10]. Patients with a specific medical history and at high risk of medication errors could potentially benefit from this study.

In the last few decades, machine learning (ML)-based computational tools and in silico models to predict ADMET (Adsorption, Distribution, Metabolism, Excretion, and Toxicity) profiles of molecules have been increasingly integrated into the drug discovery process in order to mitigate late-stage failures caused by poor pharmacokinetics and toxicity. Especially, Quantitative Structure Activity Relationships (QSAR) methods can be used to predict the toxicity quantitatively. “ADMET design” is a paradigm where ADMET properties and biological efficacy have an equally important value in initial phases of drug discovery. In silico approaches can be used as a multidimensional search and optimization tool for incorporating multiple variables and for using the relevant experimental data in the most effective manner. ML approaches, including such as ANNs (Artificial Neural Networks) [11] or SVMs (Support Vector Machines) [12] to QSAR/QSPR modeling [13] can be used to calculate ADMET properties. For QSAR/QSPR modeling, the inputs to mathematical prognostic models comprise the chemical structures that are encoded by calculated values of molecular descriptors. ANN models need an extensive experimental database to be trained and have become more commonly employed in drug discovery starting in the late 1990s and are currently considered to be better predictors compared to other models  [14]. To improve the accuracy of these computational predictions, ANN Ensemble (ANNE) or SVM Ensemble (SVME) approaches may be employed by training several ANNs or SVMs and using the ensemble average of their outputs [15].

In the following sections, we cover a large range of toxicities including cardiotoxicity, hepatotoxicity, endocrine, carcinogenicity and sensitivity paving the way to to optimize the choice of a medication that may currently be approved, for example, a patient with a specific risk factor such as heart disease should avoid the drugs that cause cardiotoxicities. We also used models for metabolite prediction to have a better understanding of drug toxicities and adverse drug interactions.

## Materials and methods

### Materials

We used selected potential antiviral molecules from two different studies as follows.

#### 69 compounds

We adopted 69 existing FDA-approved drugs, drugs in clinical trials and/or preclinical compounds, which are currently being evaluated for efficacy in live SARS-CoV-2 infection assays, as reported in [10]. These molecules are derived with expert analysis of human protein interactors of SARS-CoV-2 and reagents and drugs that modulate them; and they are not currently available in the chemoinformatically-searchable literature. In their study, the authors performed cloning, tagging, and expressing of 26 of the 29 viral proteins in human cells and were able to identify the human proteins that are physically associated with these vital proteins using affinity purification mass spectrometry (AP-MS) assays, which identified 332 high confidence SARS-CoV-2-human PPIs (protein-protein interactions). The authors identified 67 druggable human proteins or host factors targeted by 69 existing FDA-approved drugs, drugs in clinical trials and/or preclinical compounds. Identifying the host dependency factors facilitating virus infection may reveal important and deeper understanding of effective molecular targets to develop antiviral therapeutics that broadly act against SARS-CoV-2 and other deadly coronavirus strains.

#### 21 compounds

We used 21 known drugs that have been shown [9] to exhibit dose–response relationships out of 100 molecules inhibiting viral replication of SARS-CoV-2. One hundred molecules were selected from nearly 12,000 drugs that are either in clinical-stages of development or FDA-approved small molecules to identify candidate therapeutic drugs for COVID-19. Thirteen of these 21 drugs exhibit effective concentrations equivalent to probable achievable therapeutic doses in patients, such as PIKfyve kinase inhibitor apilimod [16] and the cysteine protease inhibitors MDL-28170, Z LVG CHN2, VBY-825 and ONO 5334. It is found that MDL-28170, ONO 5334 and apilimod antagonize viral replication in human pneumocyte-like cells derived from induced pluripotent stem cells. It has also been observed that apilimod showed antiviral efficacy in a primary human lung explant model.

### Methods

We used two different methods in our calculations: (1) in silico ADMET modeling employing ADMET predictor (Simulation Plus) software to calculate ADMET properties, toxicity and risks of the compounds under study, and (2) QSAR machine learning (ML) based modeling using a software written by our group to predict drug blockade of the hERG1 channel.

### In silico ADMET modeling using ADMET Predictor software and models

**Fig. 1:**
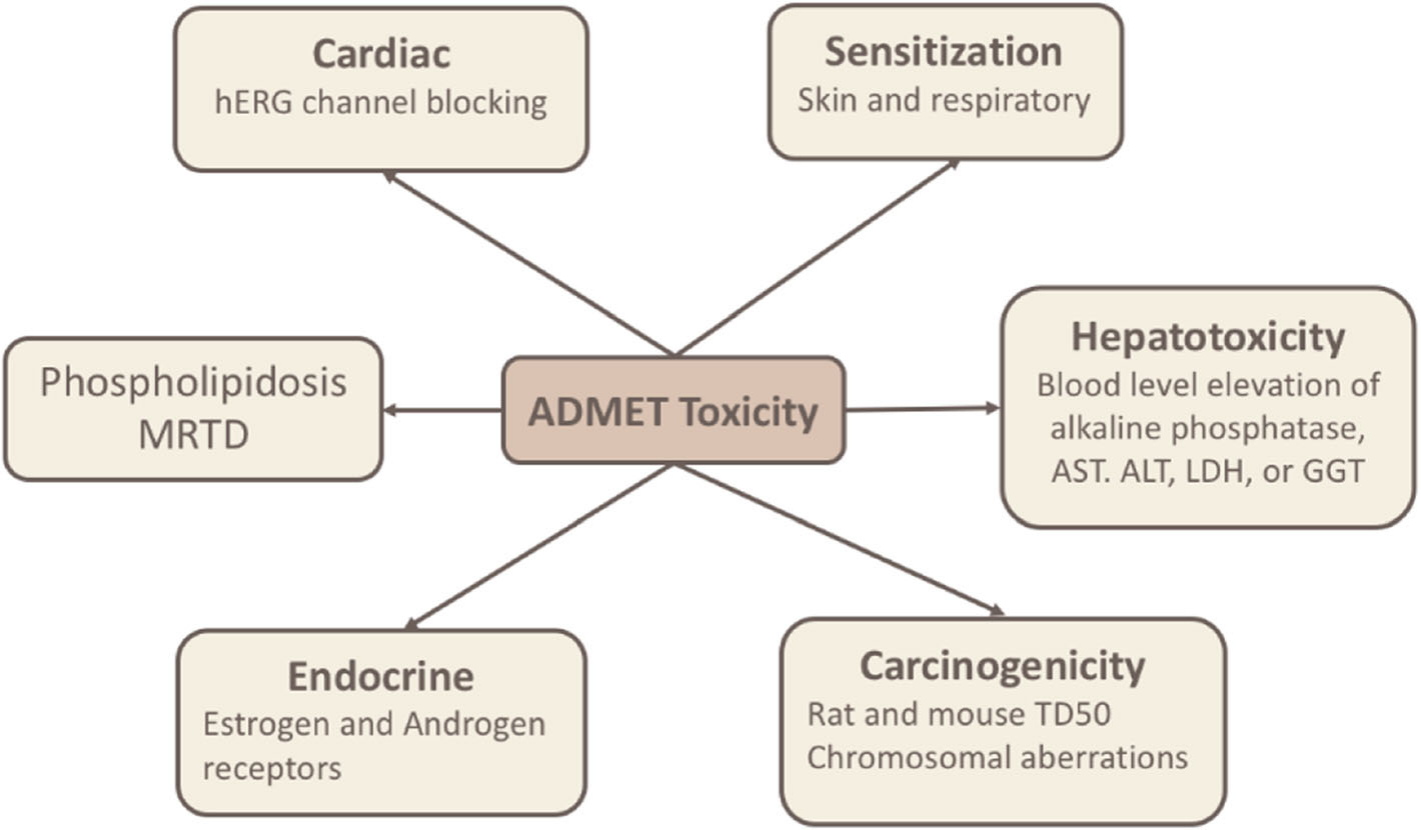
Models in ADMET Predictor’s Toxicity Module

ADMET Predictor (https://www.simulations-plus.com/software/admetpredictor/) is used to construct ANN and SVM classification QSAR models to predict ADMET parameters of compounds. In general, in silico models can be described in terms of how a particular biological endpoint being predicted is defined, the kind of descriptors used to characterize the molecular structure, and the mathematical formulation used to associate the descriptors to the model output. ADMET Predictor accepts 2D/3D-dimensional SDF files of the structures of the compounds as input. Then, compounds can be mathematically codified to more than 350 molecular descriptors. Molecular descriptors are used to create prediction models using statistical approaches or machine learning techniques such as support vector machine (SVM) and the artificial neural network (ANN). Consequently, the generated model is used to predict the corresponding properties of new compounds. ADMET Predictor’s Toxicity Module is a statistical model which relies on molecular descriptors. Statistical models are typically better able to provide a more quantitative indication of how reliable a particular prediction will be, i.e., of how confident one can be in it [17]. The ‘bottom-up’ approach taken in ADMET Predictor creates a separate model for each of the ten constituent assays, then combines the results into an overall toxicity risk. The default behavior is to flag a compound as probably ie. mutagenic/metabolic/toxic in the aggregated output. Various toxicity properties of the compounds including cardiac, hepatotoxicity, endocrine, carcinogenicity and sensitivity can be predicted in silico using the toxicity module of ADMET Predictor (version 9.5, Simulation Plus, Lancaster, CA, USA) software (see Fig. 1) [18, 19].

In silico methods and software that is commonly used to predict a compound’s properties comes with two qualitative estimations rather than being limited to the absolute terms: (1) an estimate of the reliability of that prediction (2) an indication of how that compound plausibly resembles the compounds that are used to train the model. Details about training set sizes, data sources, plots of predicted versus observed values are thoroughly presented in the manual documentation provided for  ADMET Predictor users [20]. A compound is considered ‘out of scope’ if it falls outside of the model’s ‘applicability domain.’ Predictions for out-of-scope compounds are flagged in ADMET Predictor’s output. In our analysis none of the selected compounds were flagged as out of scope which shows that the applicability domains of the training and test data sets overlap. Classification confidence estimate for different models for our selected compounds was mostly above 90%. For a more detailed description of predictive certainty of ADMET Predictor’s toxicity and metabolism modules, we refer the reader to an excellent publication by RD. Clarck [21].

In the following sections, we discuss in detail the toxicity and risk models related to toxicity models of ADMET Predictor software.

### Allergic skin and respiratory sensitization

A compound or substance that stimulates dermal allergic reactions is referred to as a skin sensitizer. TOX_SKIN model employed the murine local lymph node assay (LLNA) which has been established to be a successful tool in evaluating the relative potency of compounds as skin sensitizers for assessing the associated risks (https://ntp.niehs.nih.gov/whatwestudy/niceatm/index.html). Recently, this model has been endorsed for calculating the relative effectiveness of skin sensitizing chemicals. We also report here the results of the TOX_RESP model [19, 20], which indicate respiratory sensitization (see Table 1).

**Table 1:**
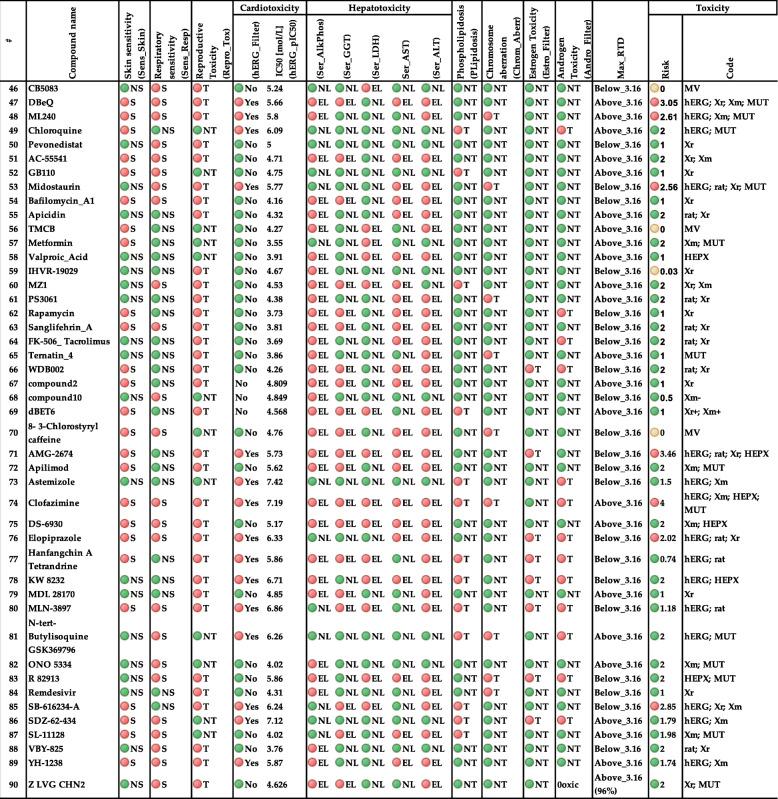
Predicted toxicities and toxicity risk for 90 compounds performed by ADMET Predictor software. ADMET Predictor identifier of each toxicity is mentioned in the parenthesis (see abbreviation list). In particular, Tox_hERG_Filter is a qualitative estimation of the affinity to the hERG potassium channel in human and Tox_hERG is the affinity to the hERG potassium channel in human expressed as pIC50 in mol/L. Compounds with an IC50 less than or equal to 10 μmol/L were labeled Toxic (T, red), while those greater than 10 μmol/L are considered non-toxic (NT, green). Human liver adverse effect (the likelihood of causing elevation in the levels of AlkPhos, GGT, LDH, AST amd ALT enzymes) is also summarized in the hepatotoxicity section and color coded as EL (Elevated, red), NL (Normal, green). Other toxicity assessments are mentioned as Skin sensitivity, Respiratory sensitivity, Reproductive toxicity, Phospholipidosis, Chromosome aberration, Estrogen and AndrogenToxicity and Max_RTD (Maximum Recommended Therapeutic Dose). The abbreviations used are Nonsensitive (NS, green), Sensitive (S, red), EL (Elevated, red), NL (Normal, green), T (Toxic) and NT (Nontoxic). Toxicity risk (possible range 0–7) is the risk connected with predicted toxicity problems a compound might have. Toxicity risk less than 2 is considered as safe (green). Check rules and abbreviations for TOX_Risk and ToX_Code in the risk section for the codes. MV stands for MISSING_VALUE in ADMET predictor and color coded in yellow

### Reproductive toxicity

Reproductive toxicity is an essential regulatory endpoint that is categorized as developmental toxicity. Reproductive toxicity refers to any parameter that disrupts organisms’ reproductive means, such as unfavorable effects on sexual organs, performance, ease of conception, as well as any developmental toxicity experienced by the offspring. ADMET predictor used the data from the FDA/TETRIS database, which was collected originally from the literature. The qualitative evaluation of the reproductive toxicity (TOX_REPR) model [20] is presented in Table 1. Compounds are classified as either toxic “T, red” or nontoxic “NT, green” (see Table 1).

### Cardiac toxicity (affinity for hERG-encoded potassium channels)

Cardiovascular diseases continue to be a leading cause of morbidity and mortality. Medicines can significantly contribute to the high burden of cardiovascular risk factors and thus deserve special attention. The human Ether-a-go-go Related Gene (hERG) is a gene that encodes potassium channels, which mediate repolarization of the ion current in the cardiac action potential. Blockade of the transmembrane influx of K^+^ ions, and inhibition of channel trafficking in heart cells caused by drugs can lead to life-threatening ventricular arrhythmias [22–24]. The ADMET toxicity module uses two neural network models to assess Covid-19 drugs that may induce distinct cardiovascular toxicity by blocking the hERG channel, TOX_hERG_Filter, and TOX_hERG [20]. The first one can be considered as a classification model which determines if the compound is expected to have an affinity for the hERG K^+^ channel. The results for TOX_hERG Filter are presented in Table 1. Compounds with their IC50 values below 10 μM are shown as “T, red” (Toxic), while those greater than 10 μM are labeled “NT, green” (Nontoxic). If a compound is predicted to be “T”, it will likely block the hERG channel. The IC50 value of the compound is predicted in molar units and shown as pIC50 (−log(IC50 [M]). The model’s performance is shown in Table 1.

As we discussed above, ADMET Predictor uses two ANNs, a classification model, and a regression model to evaluate the probability of blocking the hERG channel by a given compound. In this paper, we also used the QSAR/ML model that we recently developed to assess the possibility of blocking the hERG channel by the compounds under study (see Tables 1 and 4).

### Hepatotoxicity (human liver adverse effects)

It has been over three decades since reports on adverse effects of drugs on human livers have been accumulated by the US FDA CDER (Center for Drug Evaluation and Research). Two databases developed using this work, the Spontaneous Reporting System (SRS) and the Adverse Event Reporting System (AERS), are used by a software package called ADMET Predictor to model hepatotoxicity of many popular pharmaceuticals. Using this procedure, five separate models that correspond to individual liver enzymes used in hepatotoxicity diagnostics are obtained:

alkaline phosphatase (Ser_AlkPhos) increase, gamma-glutamyltransferase (Ser_GGT) increase, lactate dehydrogenase (Ser_LDH) increase, aspartate aminotransferase (Ser_AST) increase, and alanine aminotransferase (Ser_ALT) increase [20]. Compounds classified as elevated enzymes level “EL, red” and normal enzymes level “NL, green” are shown in Table 1.

### Phospholipidosis

In individuals, lysosomal storage disorders can cause the accumulation of phospholipids in the tissue and body instead of regular metabolism by lysosomes. Lysosomes are defined as cellular organelles carrying specific enzymes that metabolize waste materials to promote their elimination. The origin of metabolic disorders can be either hereditary or drug-induced, as the latter manifests in phospholipidosis. Phospholipidosis is regarded to have a significant role in the nervous system. When present, phospholipids may cause disorder in neuronal cell signaling leading to various genetic diseases, e.g. Niemann-Pick disease. In drug discovery, the process of drug development may be delayed or halted due to the identification or as a result of extra testing if needed to satisfy the obligations of regulators. ADMET Predictor develops a classification model named TOX_PHOS [20] by utilizing a data set of chemicals with a known phospholipidosis profile obtained from the literature. Overall, electron microscopy was used to identify all non-inducers and some inducers while information about the presence of foamy macrophages or vacuolations was used to detect the rest of the inducers. In Table 1, non-inducers are labeled as Nontoxic “NT, green”, whereas inducers are labeled as Toxic “T, red”.

### Chromosomal aberrations

An ANN ensemble model named TOX_CABR provided by ADMET Predictor [20] is used to assess the genotoxic potential of chemicals and drugs. A training data set with observed CA results that exhibit a very balanced distribution of Toxic “T” and Nontoxic “NT” is used for this ANN ensemble model. Compounds classified as toxic “T, red” and nontoxic “NT, green” are shown in Table 1.

### Acute rat toxicity

The acute rat toxicity model, referred as TOX_RAT [20], is built on the amount of an orally administered chemical substance (in mg per kg of body weight) that resulted in lethality of half of the rats in a given study. The grand challenge to build a QSAR model is the permanence of such a diverse dataset. ADMET predictor utilizes the data from the following resources: Registry of Toxic Effects of Chemical Substances data set, referred as RTECS, (the version associated with the CDC’s NIOSH), and the ChemIDplus database. The unit used for LD50 in TOX_RAT model is mg/kg. Compounds with the predicted toxicities given by LD50 (mg/kg) are shown in Supplementary Table 1. According to the risk criteria in risks section, (acute toxicity in rats, ra: TOX_RAT < 300) is considered as high risk. Supplementary Table 1 is presented in a color-coded fashion such that the most dangerous drugs are shown as red, while safe drugs are shown in green (see Supplementary Table 1).

### Endocrine toxicity

Drug compounds compete with sex hormones to inhibit and interact with the estrogen and/or androgen receptors, which can drive disruptions in endocrine system signaling, such as blocking the passage of standard hormonal signals and causing toxicity. Androgens, for instance, play a significant role in developing and maintaining the male phenotype and the pathology and treatment of prostate cancer.

ADMET Predictor uses two models for predicting endocrine toxicity by qualitatively assessing estrogen receptor toxicity in rats (TOX_ER_Filter) and androgen receptor toxicity in rats (TOX_AR_Filter) [20]. Qualitative estimation of androgen and estrogen receptor toxicity in rats is shown in Table 1 as NT ‘Nontoxic’ and T ‘Toxic’ (see Table 1).

### Maximum recommended therapeutic dose

US FDA’s CDER has collected a maximum recommended therapeutic dose (MRTD) database to shed light on the relationship between structure, toxicity, and no-effect level (NOEL) of chemicals in humans to assess the health-related effects. ADMET Predictor utilizes ANN Ensemble models to predict the MRTD for compounds in mg/kg-BodyWeight/day units. When the prediction is higher than 3.16 mg/kg-BW/day, it is indicative of an “inactive” (green color-coded) compound with improbable side effects, and estimations less than 3.16 are labeled with red color with significant potential for side effects. The relevant results for MRTD are presented in Table 1.

### Chronic carcinogenicity and mutagenicity

ADMET Predictor adopted Carcinogenic Potency Database (CPDB) is made available by the EPA’s DSSTox program to develop two quantitative chronic carcinogenicity and mutagenicity models: Rat_TD50 and Mouse_TD50. Rat_TD50 predicts the TD50 value of a selected compound. The TD50 is the dose of a substance given to rats orally throughout their lifetimes resulting in half of the population experiencing tumors. Furthermore, Mouse_TD50 predicts the TD50 value in mice. Both models predict TD50 values in units of mg/kg/day. According to the risk criteria in section 2.1.12.1 (carcinogenicity in chronic mouse studies, Xm: Mouse_TD50 < 25) and (carcinogenicity in chronic rat studies, Xr: Rat_TD50 < 4) are considered as high risk. Table S1. is color-coded as most dangerous drugs are shown in red, while safe drugs are shown in green, respectively (see Supplementary Table 1).

The outcome of 10 models estimating Ames Mutagenicity in five different strains of Salmonella with or without metabolic activation (m labeled) is summarized in Table S1. Developed by Ames et al. using strains of the *Salmonella typhimurium* as a time and cost-effective option for testing in rodents, the Ames Mutagenicity measures the mutagenic potential of chemical compounds. The 10 ANN Ensembles featured with TOX_MUT* are qualitative models that are used to predict the mutagenicity of chemical compounds either as “+” (i.e., mutagenic) or “-” (otherwise).

#### Risks

##### Mutagenicity risk

ADMET Predictor summarizes the output of mutagenicity models employing ADMET Risk and ADMET Code for mutagenicity in *S. typhimurium* (MUT_Risk and MUT_Code), depicting the results of “virtual Ames testing.” There are ten TOX_MUT models, which individually take part in the assessment of the mutagenicity anticipated for five strains of *Salmonella typhimurium* with and without microsomal activation (e.g., TOX_MUT_102 and TOX_MUT_m102). Risk code and criteria for mutagenicity are listed as below:

- S1: (TOX_MUT_97 + 1537 = “+”)
- m1: (TOX_MUT_m97 + 1537 = “+” AND NOT TOX_MUT_97 + 1537 = “+”)
- S2: (TOX_MUT_98 = “+”)
- m2: (TOX_MUT_m98 = “+” AND NOT TOX_MUT_98 = “+”)
- S3: (TOX_MUT_100 = “+”)
- m3: (TOX_MUT_m100 = “+” AND NOT TOX_MUT_100 = “+”)
- S4: (TOX_MUT_102 + wp2 = “+”)
- m4: (TOX_MUT_m102 + wp2 = “+” AND NOT TOX_MUT_102 + wp2 = “+”)
- S5: (TOX_MUT_1535 = “+”)
- m5: (TOX_MUT_m1535 = “+” AND NOT TOX_MUT_1535 = “+”)
- SU: (TOX_MUT_97 + 1537 = Undecided OR TOX_MUT_98 = Undecided OR TOX_MUT_100 = Undecided OR TOX_MUT_102 + wp2 = Undecided OR TOX_MUT_1535 = Undecided (weight = 0.5))
- mU: (TOX_MUT_m97 + 1537 = Undecided OR TOX_MUT_m98 = Undecided OR TOX_MUT_m100 = Undecided OR TOX_MUT_m102 + wp2 = Undecided OR TOX_MUT_m1535 = Undecided (weight = 0.5))

MUT Risk rule codes for mutagenicity from Table 3 are as follows: Risk of positive Ames test results with (m*) or without (S*) microsomal activation for *Salmonella typhimurium* strains, where * = TA97 or TA1537; TA98; TA100; TA102 or WP2 uvrA strain of *E. coli*; TA1535, respectively. NIHS panel predictions are not separated with respect to S9 activation or lack thereof.

The results related to the mutagenicity risk are presented in Table 2. We highlighted all the compounds with MUT_Risk 2 or higher as red.

**Table 2:**
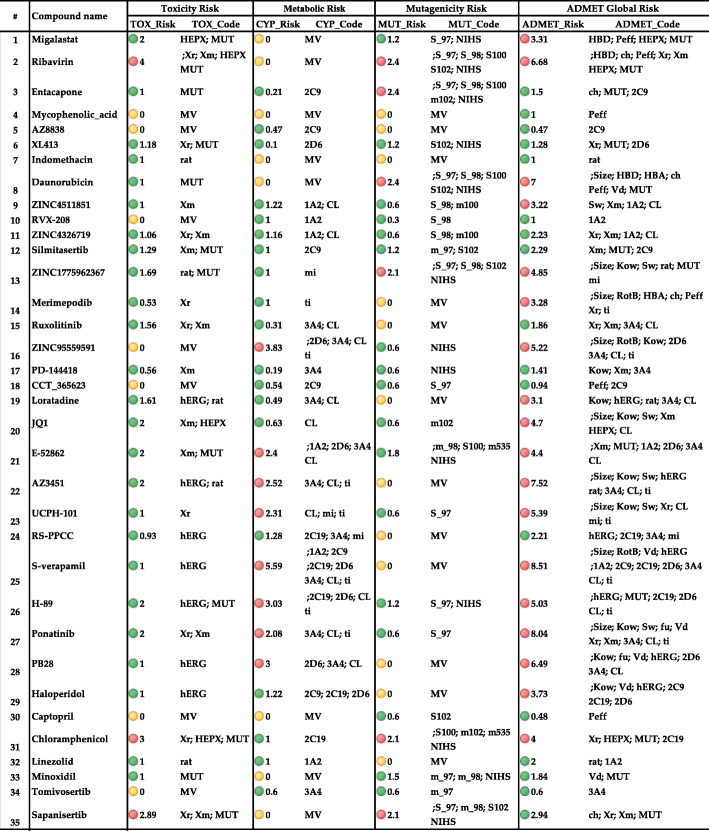
ADMET Risk and ADMET Code for toxic liability, mutagenicity in *S. typhimurium* liability, metabolic liability, and the global ADMET Risk and ADMET Code summarizing all other ADMET Risk/Code models. TOX_Risk (possible range 0–7) and TOX_MUT_Risk (possible range 0–11). Check codes, rules and abbreviations for in risk section. The high risk values were highlighted with red (TOX_Risk> 2, CYP_Risk > 2, MUT_Risk > 2 and ADMET_Risk > 3), while the acceptable values are highlighted with green. MV stands for MISSING_VALUE in ADMET predictor and color coded with yellow

##### Toxicity risk

ADMET Risk and ADMET Code for toxic liability are TOX_Risk and TOX_Code, respectively. The TOX_Risk model includes seven rules, each of which has an associated weight of one. Risk code and criteria for potential hERG liability, acute toxicity in rats, carcinogenicity in chronic rat studies, carcinogenicity in chronic mouse studies, hepatotoxicity and SGOT and SGPT elevation are as follows respectively:

- hERG = (TOX_hERG > 6)
- ra = (TOX_RAT < 300)
- Xr = (Rat_TD50 < 4)
- Xm = (Mouse_TD50 < 25)
- Hepatotoxicity = (Hp: (TOX_AlkPhos = Toxic OR TOX_GGT = Toxic OR TOX_LDH = Toxic) AND (TOX_SGOT = Toxic OR TOX_SGPT = Toxic))
- SGOT and SGPT elevation = (SG: TOX_SGOT = Toxic AND TOX_SGPT = Toxic)
- Mu = (TOX_MUT_Risk > 2)

The possible value range for TOX_MUT_Risk is 0–11 and it is 0–7 for TOX_Risk.

The results related to the toxicity risk are presented in Table 2. We highlighted all the compounds with TOX_Risk 2 or higher as red.

##### Metabolism risk

Metabolism module of ADMET predictor featured CYP_Risk model encompasses seven rules, each with a weight of 1.“Substr” stands for the expectation of being substrate for certain isoenzyme. “Clint” means intrinsic clearance constant for this isoenzyme. Ki_Mid and Ki_tes are inhibition constants for Midazolam and testosterone, (see List of Abbreviations and Table 3). The code and criteria (being excessive CYP_(1A2, 2C19, 2C9, 2D6, 3A4 clearance) as well as Ki_Mid and Ki_tes for metabolism risk is presented in the following paragraph.

- 1A2 = (CYP_1A2_Substr = Yes AND MET_1A2_CLint > 30)
- 2C19 = (CYP_2C19_Substr = Yes AND MET_2C19_CLint > 30)
- 2C9 = (CYP_2C9_Substr = Yes AND MET_2C9_CLint > 30)
- 2D6 = (CYP_2D6_Substr = Yes AND MET_2D6_CLint > 30)
- 3A4 = (CYP_3A4_Substr = Yes AND MET_3A4_CLint > 30)
- Mi = (MET_3A4_Ki_Mid < 1.5 AND (MET_3A4_I_mid = Yes OR MET_3A4_Inh = Yes))
- Ti = (MET_3A4_Ki_tes < 1.0 AND (MET_3A4_I_tes = Yes OR MET_3A4_Inh = Yes))

**Table 3:**
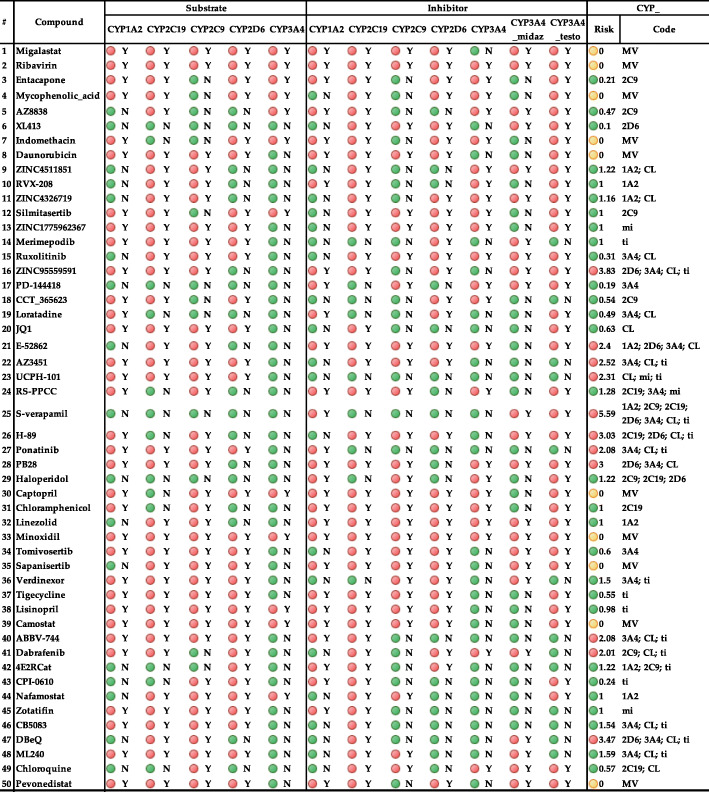
Block1: Substrate Classification Models for Cytochrome P450 1A2, 2C9, 2C19, 2D6, and 3A4. Block 2: Inhibition models for cytochrome P450 1A2, 2C9, 2C19, 2D6, and 3A4 (qualitative es-timation), as well as a specific inhibition of the CYP 3A4-mediated metabolism of midazolam and testosterone. We labeled Y if a given chemical structure is a substrate for P450 isozymes and (N) if it is not a substrate for P450 isozymes. ADMET Risk and ADMET Code for metabolic liability are CYP_risk and CYP_code, respectively. Metabolic risk greater than 2 is highlighted with red, while the acceptable values are highlighted with green. Cyp risk rules and codes are described in the risk section

CYP_Risk is 2 or greater for a little over 10% of the compounds in the focused World Drug Index (WDI). We highlighted all compounds with CYP_Risk 2 or higher as red.

##### ADMET global risk

Eventually, the ADMET predictor recapitulates the main outcomes and generates a global

classification (ADMET_Risk). The global ADMET_Risk itself combines the rules from S + Absn_Risk, CYP_Risk, TOX_Risk and additional two rules for low fraction unbound in plasma and high steady-state volume of distribution. Codes and criteria for the additional rules are as follows, respectively.

- fu = (S + PrUnbnd < 3.5%)
- Vd = (S + Vd > 5.5)

There are then 24 different rules that contribute to the ADMET_Risk model. Full ADMET Risk rule codes are mentioned in abbreviations section (see Table 2). We highlighted all the compounds with ADMET_Risk 3 or higher as red.

#### Cytochrome P450 enzymes (CYPs) model

ADMET Predictor engages different classification models such as substrate, SoM, and kinetic predictions for different isoforms of CYP to predict the metabolites that are more probable to occur. Finally, ADMET can estimate the contribution each will make to CYP metabolism in vivo. ADMET Predictor built nine classification QSAR models from literature data for the following UGT isozymes responsible for Phase II drug metabolism: UGT1A1, UGT1A3, UGT1A4, UGT1A6, UGT1A8, UGT1A9, UGT1A10, UGT2B7, and UGT2B15. These models predict whether a compound will be metabolized by one or more of these enzymes.

#### QSAR machine learning (ML) model to predict drug blockade of hERG1 channel

This model is based on the eXtreme gradient boosting (XGBoost) algorithm [25]. Briefly, molecular and pharmacophoric descriptors (float, integer and binary values) were generated from each compound’s SMILES string using RDKit open source toolkit for chemoinformatics [26]. These descriptors were used as input for the prediction of inhibitory potency for each compound (pIC50 of inhibition). The model also provides metrics to assess the compliance with its applicability domain (AD = True/False) in terms of the Minimum Distance to Training set (MDT) that is based on the Tanimoto similarity to the compounds used in the training set of the model. pIC50 has its regular meaning – a large positive number is equivalent to high affinity blockers. The potentially dangerous compounds are in the range between 5.5 to +infinity. Since it is an ML model, we also report the applicability domain as measured by the similarity matrix and distance to the training set. What it means, is that many compounds have a unique scaffold not present in the model. We currently use the receptor map models (SILCS) to obtain somewhat more realistic estimates. That is, if AD (applicability domain) is “False”, the confidence in pIC50 prediction is low. This is a well-known issue with any QSAR/ML models facing unknown molecular scaffolds.

## Results

### In silico prediction of toxicity and ADMET properties

Various Toxicity and ADMET-related properties of the investigated compounds were predicted in silico using the toxicity module of ADMET Prediction™ (version 9.5, Simulation Plus, Lancaster, CA, USA) software, where a broad range of toxicities are covered including cardiac, hepatotoxicity, endocrine, carcinogenicity and sensitivity (see Fig. 1) [18, 19].

Several toxicity parameters are used for the evaluation: 1. allergenic skin sensitization (TOX_SKIN), 2. allergenic respiratory sensitization (TOX_RESP), 3. reproductive Toxicity (Repro_Tox), 4. cardiotoxicity (TOX_hERG_Filter), 5.Cardiotoxicity IC50 [mol/L] (TOX_hERG), 6. hepatotoxicity (five liver enzymes elevations: Ser_Alkphos, Ser_GGT, Ser_LDH, Ser_AST, Ser_ALT) 7. phospholipidosis (PLipidosis), 8. chromosome aberration (Chrom.Aberr),9. acute toxicity in rats (Rat_Acute), 10. carcinogenicity toxicity in rat (Rat_TD50), 11. carcinogenicity toxicity in mouse (Mouse_TD50), 12. estrogen (Estro_Filter) and androgen (Andro_Filter) binding, 13. maximum recommended therapeutic dose (Max_RTD). Among the 90 compounds evaluated here, none of the ligands showed toxicity for the selected parameters whereas the remaining ligands exhibited toxicity for only a few parameters (See Table 1). Additional details for each model and risks are presented in the Materials and Methods section.

Compounds with the predicted acute toxicity in rats, LD50 (mg/kg), are shown in Supplementary Table 1. Supplementary Table 1 is presented in a color-coded fashion such that the most dangerous drugs with (acute toxicity in rats, ra: TOX_RAT < 300) are shown as red, while safe drugs (acute toxicity in rats, ra: TOX_RAT > 300) are shown in green (see Supplementary Table 1) [20].

The results related to the mutagenicity risk (MUT_Risk 2 or higher as red), toxicity risk (TOX_Risk 2 or higher as red), metabolic risk (CYP_Risk is 2 or greater as red) and Full ADMET Risk (ADMET_Risk 3 or higher are highlighted as red.) is presented in Table 2. All risk rule codes are mentioned in the abbreviations section. MUT_Risk, described in the risk section, predicts overall mutagenicity by adding instances of “+.” The results related to mutagenicity are presented in Supplementary Table 2 [20].

#### In silico study of cytochrome P450 enzymes (CYPs) to understand drug-drug interactions (DDI)

In silico tools are broadly used to predict substrates and inhibitors of metabolic enzymes and sites of metabolism in molecules where the metabolic reaction occurs. These predictions facilitate the multidimensional drug discovery procedure, paving the way to fulfill the stability, enhancements of in vivo half-life, and circumventing the toxic metabolites. The most important enzymes in Phase I metabolism belong to the cytochrome P450s family (CYPs) since they provide the most first-generation metabolites and have a high proportion of toxic/reactive metabolites. They are a family of heme-containing enzymes where at least 57 CYP isoforms have been authenticated in humans. Changes in the CYP enzyme activity can influence the metabolism and clearance of drugs, therefore, the inhibition of cytochrome P450 is the most prominent cause of drug toxicities. Substrate Classification Models for Cytochrome P450 1A2, 2C9, 2C19, 2D6, Inhibition models for cytochrome P450 1A2, 2C9, 2C19, 2D6, and 3A4 (qualitative estimation), as well as a specific inhibition of the CYP 3A4-mediated metabolism of midazolam and testosterone are presented in Table 3. We labeled it Y if a given chemical structure is a substrate for P450 isozymes and (N) if it is not a substrate for P450 isozymes. ADMET Risk and ADMET Code for metabolic liability are CYP_risk and CYP_code, respectively.

The uridine 5′-diphosphate-glucuronosyltransferases (UGT) enzymes are distributed in various organs in the human body and abundantly expressed in the liver as the central metabolic organ. The UGT enzymes catalyze in Phase II metabolism through glucuronidation, the primary Phase II metabolic pathway, which leads to a more straightforward clearance of xenobiotics. UGT enzymes in humans are predominantly created by the liver except UGTs 1A8 and 1A10 produced by the gastrointestinal tract. The probability of metabolism by human uridine 5′-Diphosphate-Glucuronosyltransferases (UGT) is summarized in Supplementary Table 3 [27].

### Qualitative and quantitative prediction of drug blockade of hERG1 channel based on QSAR machine learning (ML) model

The cardiotoxicity potential of the compounds’ datasets listed in Table 4. was assessed using our recently reported machine learning algorithm for the prediction of drug-induced blockade of hERG channel (pIC50 of inhibition) [28]. The results of this method are in agreement with the results of ADMET Predictor software for cardiotoxicity, except for a few compounds that are not among the compounds we selected in the discussion section. For a predictive classification model, applicability domain (AD) identifies the chemical space where the model’s predictions are deemed reliable. As can be seen from Table 4, all compounds used in this study analyzed with QSAR ML model fit in the applicability domain, which is represented as ‘TRUE’ under the ‘AD’ column. Details about the metrics and scores used to estimate the model performance of the QSAR model can be found in Wacker and Noskov’s paper [28].

**Table 4:**
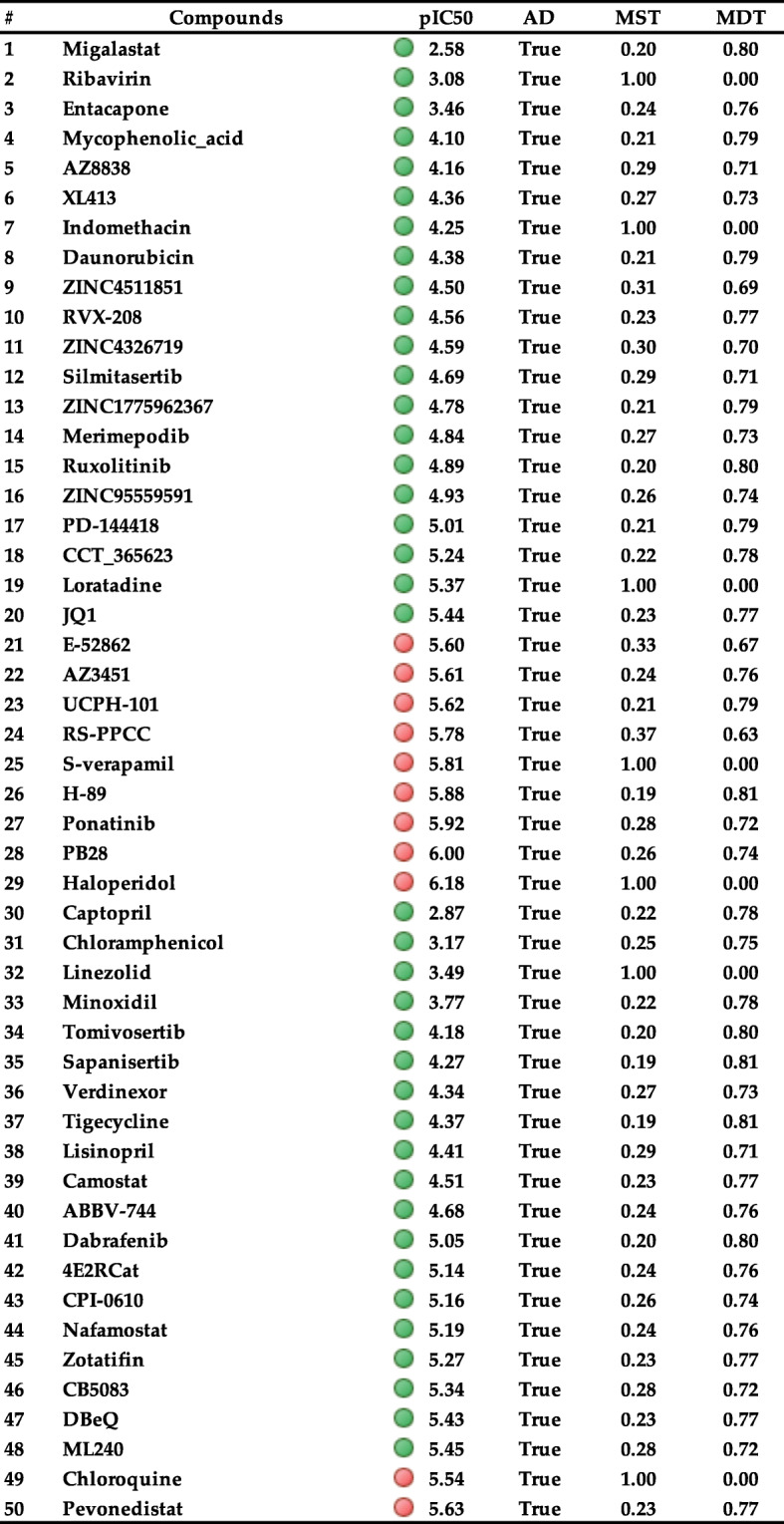
Predicted affinity to the hERG potassium channel in human expressed as pIC50 in mol/L using our ML model. (Abbreviations: AD: Applicability domain, MST: Similarity matrix and MDT: Minimum Distance to Training). NA stands for not available

## Discussion

One of the major dangers faced by COVID-19 patients is respiratory failure accompanied by cardiovascular complications with extensive endothelial dysfunction and severe inflammation. ACE-2 receptors are the cell-entry gateway for SARS-CoV-2. Drugs that passed different filters used in the present study, such as (ADMET Global Risk = < 3 & Cardiotoxicity = NT and Respiratory toxicity = NS) are Entacapone, Indomethacin, Captopril, Linezolid, Valproic_Acid, AZ8838, Tomivosertib, TMCB, Ternatin_4, and MDL 28170. Among them only Entacapone, Indomethacin, Captopril, Linezolid, Valproic_Acid are FDA approved drugs and hence can be used in an off-label mode with some caution but they appear to exhibit much greater safety profiles than the rest of the panel. A brief summary of the final compounds is as follows.

### Entacapone

Entacapone [29, 30], a drug commonly used to reduce the signs and symptoms of Parkinson’s disease (https://go.drugbank.com/drugs/DB00494). A member of nitrocatechols class, Entacapone is a selective and reversible COMT (catechol-O-methyl transferase) inhibitor. In the management of the motor complications seen in Parkinson’s disease, Entacapone is administered with levodopa/carbidopa in patients with wearing-off symptoms. Even though Entacapone is related to tolcapone structurally and pharmacologically, it is not associated with hepatotoxicity unlike tolcapone. Entacapone is available for administration as oral tablets. As of now, there is no research addressing the possibility of repurposing this drug for Covid-19.

### Indomethacin

Indomethacin [31] is a well-known non-steroidal anti-inflammatory drug (NSAID) and widely utilized for treating osteoarthritis, rheumatoid arthritis, ankylosing spondylitis, acute shoulder pains, and acute gouty arthritis. Indomethacin relieves muscle pain and reduces fever, swelling, and tenderness by quelling inflammation in rheumatoid arthritis (https://go.drugbank.com/drugs/DB00328). Consisting of structurally unrelated agents, Indomethacin is classified chemically as an indole-acetic acid derivative with the chemical name 1- (p-chlorobenzoyl)25-methoxy-2-methylindole-3-acetic acid [32]. First approved by the FDA for use in the U.S. in the mid-1960’s, Indomethacin has been extensively investigated in clinical trials as an effective NSAID for blocking prostaglandin synthesis as well as for treating headaches and migraine. Indomethacin is available as oral capsules and other administration methods, including rectal and intravenous procedures. The pharmacological effect of indomethacin is not completely clear; however, it is believed that it can cooperate through nonselective inhibition of the enzyme cyclooxygenase (COX), the primary enzyme in charge of the catalysis of the conversion of arachidonic acid in prostaglandin and thromboxane biosynthesis.

A pre-print published recently in Medrxiv has promoted the case to use Indomethacin for relieving the symptoms in COVID-19 patients and preventing progression of pneumonia. The broad spectrum of anti-viral activity of Indomethacin, including on the SARS-Cov-2 virus, has been shown in the laboratory. It was recognized that the patients that were treated with Indomethacin progressed in recovery quantized by being afebrile sooner, and there was a nearly 50% reduction in cough and myalgia in comparison with the paracetamol set. According to this study, only one out of 72 patients with mild and moderate symptoms needed supplementary oxygen, and none of the patients with severe symptoms worsened to the degree of requiring mechanical ventilation. It was indicated that there was no proof of adverse reaction to Indomethacin, nor there was proof of renal failure or liver dysfunction [33].

In another study [34], after their preliminary results showed that cyclopentenone COX metabolites are active against a number of RNA viruses, the researchers further examined the effect of the COX inhibitor indomethacin on replication of the coronavirus. It was shown that Indomethacin is an effective inhibitor of coronavirus replication. It was recommended based on the results that since Indomethacin exhibits both anti-inflammatory and antiviral activity, it could be helpful to treat COVID-19. Interestingly, the coronavirus binding or entry into host cells are not affected by Indomethacin. Instead, the main effect of Indomethacin is preventing viral RNA synthesis at cytoprotective doses, which is independent of COX inhibition. The effectiveness of Indomethacin’s antiviral activity (more than a thousand  fold reduction in the viral yield) was validated in vivo in dogs infected with CCoV.

### Captopril

Captopril [35] is a drug that is commonly used for treating hypertension (essential or renovascular) as well as for protecting kidney function. Therefore, Captopril can be used in patients with cystinuria and other comorbidities. Captopril is regarded as a competitive inhibitor of the angiotensin-converting enzyme (ACE), which is in charge of the conversion of angiotensin I (ATI) to ATII, which is a critical element of the renin-angiotensin-aldosterone system (RAAS) that regulates blood pressure. The RAAS is a homeostatic mechanism for regulating hemodynamics, water, and electrolyte stabilizers (https://go.drugbank.com/drugs/DB01197). Captopril can be used orally in the form of tablets. Captopril is the first ACE inhibitor in the market. It is also the only ACE inhibitor with a sulfhydryl ligand that chemically bonds with cysteine, which makes it more soluble. The main connection between renin–angiotensin system (RAS) and COVID-19 is ACE2. ACE2 improves the tissue anti-inflammatory response, however it also mediates as the entry receptor for the virus [36]. The use of ACE inhibitors has been shown to deteriorate symptoms in COVID-19 patients, an observation that has become controversial [37]. It was reported by Guan et al. that most of the patients admitted with COVID-19 infection had hypertension and diabetes; nevertheless, treatment with ACE inhibitors was not examined separately [38]. In another recent study, Captopril was used as an ACE inhibitor drug to inhibit the levels of Spike protein-induced cells. They proposed that Captopril increases ACE2 levels and boosts the anti-inflammatory RAS axis in the lung. In addition, other mechanisms, such as drug-induced inhibition of ADAM17 activity, may prevent plausible up-regulation of viral entry by the drug-induced enhancement in the expression of ACE2 [35]. However, there is no clinical trial evidence of effectiveness, and little attention has been given to the possibility of toxicity caused by the use of such interventions or in what ways the balance of potential advantages and harms may differ among individuals [39].

### Linezolid

In addition to causing the disease itself, viral infections also open the way for secondary bacterial infections. These bacterial infections can be even more invasive and life-threatening than the viral infection itself. As a synthetic antibiotic, Linezolid [40, 41] is used for treating infections caused by multi-resistant bacteria including streptococcus and methicillin-resistant *Staphylococcus aureus* (MRSA). Linezolid is the first of the oxazolidinone class and it works by inhibiting bacterial protein synthesis initiation. Linezolid is absorbed quickly through oral dosing (https://go.drugbank.com/drugs/DB00601). Linezolid has been recommended as a therapy against COVID-19 [42] and is effective for treating pneumonia in COVID-19 patients according to clinical trials [43].

### Valproic acid (VPA)

As a widely-known HDAC2 inhibitor, VPA is proved to be safe for treating central nervous system diseases such as epilepsy as well as cancer and it has been in use for more than 50 years [44–46]. In humans, VPA is absorbed by endothelium, which plays a central role in inflammation, thrombosis and cardiovascular complications, almost instantly (within a minute) of intravenous injection. It is also offered as a delayed-release oral tablet with a T_max_ of 4 h [47]. The SARS-CoV-2 virus is known to use ACE-2 receptors as an entry door to infect host cells, which are mainly expressed in endothelial cells [48, 49]. Altogether, this information indicates that VPA and endothelium play a major role in COVID-19 infections Singh et al. [50].

This is an important piece of information carrying significant clinical relevance for COVID-19 as ACE-2 can be considered as a cell “entry door” for SARS-CoV-2 and because it has been demonstrated that SARS-CoV-2 infection can be intensified due to over-expression [51] and depreciated due to inhibition of ACE-2 [52–54]. Their investigation revealed that VPA can be utilized as a preventative strategy for tackling COVID-19 as it inhibits the rate of infection of SARS-CoV-2 by decreasing its receptor ACE-2 expression level. Moreover, they showed that the expression of IL-6 is declined in VPA-treated endothelial cells. This is an important finding for COVID-19 since inflammation and thrombosis, as one of the significant causes of death in COVID-19, is caused by the so-called “cytokine storm” of interleukins such as pro-inflammatory IL-6 in COVID-19 patients’ lungs [3]. Specifically, the IL-6 level can be used to predict respiratory failure in COVID-19 patients and severe lung damage can be caused by IL-6 inhibitors [55]. Endothelial cells can produce pro-inflammatory cytokines that “activate” the ECs to secrete tissue factor that controls thrombosis [56]. Remarkably, VPA also considerably reduced ICAM-1 expression, which is a marker for endothelial “activation” [57], demonstrating decrease in endothelial activation. This is an important finding as it may help develop a strategy for treating COVID-19 since inflammation and blood clotting are linked to regulatory molecules IL-6 and ICAM-1 induced by VPA. In a recent communication, Unal et al. proposed that VPA can potentially be used as a COVID-19 treatment drug [58].

In addition, recently, many drugs such as hydroxychloroquine, remdesivir, favipirapir, tocilizumab, ivermectin, dexamethasone have been reported as a potential treatment. However, most of these drugs have not been included in our study since they were not identified by the earlier work that was used by us in the toxicity prediction computations. However, according to our calculations, remedisivir, which is among the database of drugs that we studied as a potential treatment for COVID-19, has a very high ADMET risk value (about 10). Consequently, great caution should be exercised as using this drug as an off-label medication for COVID-19.

## Conclusions

This paper reports the results of using the ADMET Predictor software package to predict the toxicities associated with the aforementioned 90 compounds considered for potential off-label use to treat COVID-19. Such computational consensus models can offer enhanced prediction performance thereby providing a useful and effective tool for toxicity screening of molecules with reduced cost, time, and animal testing. The main molecular descriptor values were calculated using the 3D structures of top ligand hits. Then, the ranges of toxicity properties were predicted using mathematical models that utilize these descriptor values. Based on their non-toxic properties, five compounds were shortlisted. We suggest that these five drugs may provide therapeutic or preventative benefit to COVID-19 patients with pre-existing diseases, and offer personalized treatment in those COVID-19 patients who otherwise would be at a serious risk of life-threatening side effects. This manuscript will provide a potentially useful source of essential knowledge on toxicity assessment of 90 compounds for health care practitioners and researchers to find off-label alternatives for the treatment for COVID-19. The required drug approval time will be reduced for such use of drugs approved by FDA that demonstrated protective characteristics against COVID-19 and thus they can be tested against COVID-19 in considerably shorter time periods.

One of the strengths of this work is the choice of the compounds considered to be studied. The drugs that we studied in this manuscript are selected from an extensive set of compounds and laboratory testing methods for their efficacy but not for their potential toxicity, which is the purpose of this paper. These compounds are promising, but they may have many serious side effects, which could pose a health risk and hence require caution in prescribing them. There are limitations to this study that need to be acknowledged, potentially due to the limitations of the QSAR methodology of computational prediction, such as the volume of data or the applicability domains of different methods. To overcome these limitations, we can increase or incorporate the data volume of different QSAR models to generate a more inclusive prediction model. Also, we can utilize a consensus approach by integrating different modeling methods and then execute related predictions. If a combined model can predict the properties well, it then can be used as a consensus approach to improving ADMET prediction accuracy [59, 60].

We suggest that demonstrating the antiviral and immunomodulatory effects of these drugs with the lowest side effects would encourage clinicians to develop further clinical studies.

## Supplementary Information

**Additional file 1: Supplementary Table 1.** Toxicity models of acute toxicity in rats, Carcinogenicity toxicity in rat (Rat_TD50) and Carcinogenicity toxicity in mouse (Mouse_TD50(acute toxicity in rats, ra: TOX_RAT < 300), (carcinogenicity in chronic mouse studies, Xm: Mouse_TD50 < 25) and (carcinogenicity in chronic rat studies, Xr: Rat_TD50 < 4) is considered as high risk. **Supplementary Table 2.** Qualitative assessment of mutagenicity of the pure compound in various strains of *S. typhimurium* and *S. typhimurium*. **Supplementary Table 3.** Probability of metabolism by human uridine 5′-Diphosphate-Glucuronosyltransferases (UGT). We labeled Y if a given chemical structure is a substrate for UGT (1A1, 1A3, 1A6, 1A8, 1A9, 1A10 and 2B15) isozymes and (N) if it is not a substrate for UGT (1A1, 1A3, 1A6, 1A8, 1A9, 1A10 and 2B15) isozymes.

## Data Availability

The datasets used and analyzed in the current study are available from the corresponding author upon request.

## TOX risk rule codes

hERG: hERG inhibition
rat: Acute rat toxicity
Xr: Carcinogenicity in rat
Xm: Carcinogenicity in mice
HEPX: Hepatotoxicity
MUT: Ames positive

## CYP risk rule codes

1A2: 1A2 clearance
2C19: High 2C19 clearance
2C9: High 2C9 clearance
2D6: High 2D6 clearance
3A4: High 3A4 clearance
CL: High microsomal clearance

## Full ADMET risk rule codes

Size: Size
RotB: Rotatable bonds
HBD: H-bond donors
HBA: H-bond acceptors
ch: Charge
Kow: Lipophilicity
Peff: Permeability
Sw: Water solubility
fu: Fraction unbound
Vd: Volume of distribution
hERG: hERG inhibition
rat: Acute rat toxicity
Xr: Carcinogenicity in rat
Xm: Carcinogenicity in mice
HEPX: Hepatotoxicity
MUT: Likely Ames positive
1A2: High clearance by CYP 1A2
CL: High microsomal clearance

## Absorption risk rule codes

Size: Size
RotB: Rotatable bonds
HBD: H-bond donors
HBA: H-bond acceptors
Kow: Lipophilicity
Peff: Permeability

## Lipinski’s rule of 5 codes

Lp: Log P
Hb: Number of hydrogen bond donor hydrogens
MW: Molecular weight
NO: Number of N and O atoms
