# Supplementary material for: Computational determination of toxicity risks associated with a selection of approved drugs having demonstrated activity against COVID-19
Source: BMC Pharmacol Toxicol. 2021 Oct 21;22:61. doi: 10.1186/s40360-021-00519-5 (PMC8529228; doi:10.1186/s40360-021-00519-5)
Supplement: Supplementary file 1 — Additional file 1: Supplementary Table 1. Toxicity models of acute toxicity in rats, Carcinogenicity toxicity in rat (Rat_TD50) and Carcinogenicity toxicity in mouse (Mouse_TD50(acute toxicity in rats, ra: TOX_RAT < 300), (carcinogenicity in chronic mouse studies, Xm: Mouse_TD50 < 25) and (carcinogenicity in chronic rat studies, Xr: Rat_TD50 < 4) is considered as high risk. Supplementary Table 2. Qualitative assessment of mutagenicity of the pure compound in various strains of S. typhimurium and S. typhimurium. Supplementary Table 3. Probability of metabolism by human uridine 5′-Diphosphate-Glucuronosyltransferases (UGT). We labeled Y if a given chemical structure is a substrate for UGT (1A1, 1A3, 1A6, 1A8, 1A9, 1A10 and 2B15) isozymes and (N) if it is not a substrate for UGT (1A1, 1A3, 1A6, 1A8, 1A9, 1A10 and 2B15) isozymes. [file 40360_2021_519_MOESM1_ESM.docx]

**Supplementary Table 1.** Toxicity models of acute toxicity in rats, Carcinogenicity toxicity in rat (Rat_TD50) and Carcinogenicity toxicity in mouse (Mouse_TD50(acute toxicity in rats, ra: TOX_RAT < 300), (carcinogenicity in chronic mouse studies, Xm: Mouse_TD50 < 25) and (carcinogenicity in chronic rat studies, Xr: Rat_TD50 < 4) is considered as high risk.

**Supplementary Table 2.** Qualitative assessment of mutagenicity of the pure compound in various strains of S. typhimurium and S. typhimurium.

**Supplementary Table 2.** (Continued)

**Supplementary Table 3.** Probability of metabolism by human uridine 5’-Diphosphate-Glucuronosyltransferases (UGT)**.** We labeled Y if a given chemical structure is a substrate for UGT (1A1, 1A3, 1A6, 1A8, 1A9, 1A10 and 2B15) isozymes and (N) if it is not a substrate for UGT (1A1, 1A3, 1A6, 1A8, 1A9, 1A10 and 2B15) isozymes.

**Supplementary Table 3.** (Continued)
